# Association of sex, age and education level with patient reported outcomes in atrial fibrillation

**DOI:** 10.1186/s12872-019-1059-6

**Published:** 2019-04-05

**Authors:** Kelly T. Gleason, Cheryl R. Dennison Himmelfarb, Daniel E. Ford, Harold Lehmann, Laura Samuel, Hae Ra Han, Sandeep K. Jain, Gerald V. Naccarelli, Vikas Aggarwal, Saman Nazarian

**Affiliations:** 1 School of Nursing, Johns Hopkins University, 525 N Wolfe Street, Baltimore, MD 21205 USA; 2 School of Medicine, Johns Hopkins University, Baltimore, MD USA; 3 School of Medicine, University of Pittsburgh Medical Center, Pittsburgh, PA USA; 4 Penn State Milton S. Hershey Medical Center, State College, Hershey, PA USA; 5 University of Michigan Health System/Frankel Cardiovascular Center, Ann Harbor, MI USA; 6 School of Medicine, University of Pennsylvania, Philadelphia, PA USA

**Keywords:** Atrial fibrillation, Patient-reported outcomes, Symptoms, Quality of life, Longitudinal cohort study, Sex, Age

## Abstract

**Background:**

In atrial fibrillation (AF), there are known sex and sociodemographic disparities in clinical outcomes such as stroke. We investigate whether disparities also exist with respect to patient-reported outcomes. We explored the association of sex, age, and education level with patient-reported outcomes (AF-related quality of life, symptom severity, and emotional and functional status).

**Methods:**

The PaTH AF cohort study recruited participants (*N* = 953) with an AF diagnosis and age ≥ 18 years across 4 academic medical centers. We performed longitudinal multiple regression with random effects to determine if individual characteristics were associated with patient-reported outcomes.

**Results:**

Women reported poorer functional status (β − 2.23, 95% CI: -3.52, − 0.94) and AF-related quality of life (β − 4.12, 95% CI: -8.10, − 0.14), and higher symptoms of anxiety (β 2.08, 95% CI: 0.76, 3.40), depression (β 1.44, 95% CI: 0.25, 2.63), and AF (β 0.29, 95% CI: 0.08, 0.50). Individuals < 60 years were significantly (*p* < 0.05) more likely to report higher symptoms of depression, anxiety, and AF, and poorer AF-related quality of life. Lack of college education was associated with reporting higher symptoms of AF (β 0.42, 95% CI: 0.17, 0.68), anxiety (β 1.86, 95% CI: 0.26, 3.45), and depression (β 1.11, 95% CI: 0.15, 2.38), and lower AF-related quality of life (β − 4.41, 95% CI: -8.25, − 0.57) and functional status.

**Conclusion:**

Women, younger adults, and individuals with lower levels of education reported comparatively poor patient-reported outcomes. These findings highlight the importance of understanding why individuals experience AF differently based on certain characteristics.

## Background

Atrial Fibrillation (AF) affects an estimated 33.5 million individuals globally and is increasing in prevalence due to the aging population and obesity epidemic [1–6]. AF symptoms have been associated with increased healthcare utilization, risk of bleeding, and mortality, and decreased quality of life and functional ability [7–10]. However, the causal mechanisms of AF symptoms and reasons for great variability across patients in symptom experience are not well understood [7, 11]. Between 25 to 30% of patients with AF are asymptomatic, while other patients report severe symptoms that affect their quality of life [7, 12]. In addition, the association between AF symptoms and actual cardiac rhythm is weak [13–17]. Therapeutic management of AF symptoms is thus a complex challenge.

Despite the high variability in symptom presentation, current treatment guidelines recommend clinical treatment decisions based on a patients’ symptoms [18, 19]. AF therapies, including rate and rhythm control medications, cardioversions, and ablation, are related to improvements in symptom experience, quality of life, functional status, and emotional status [20–27]. However, the effects of therapies on symptom experience and patient-reported outcomes are variable and very little is known about the influence of individual characteristics including sex, age, and education level on these outcomes [7, 28]. Females and older adults are significantly underrepresented in AF clinical trials [29–32]. Differences in clinical outcomes by sex, age, and education from treatment are well-documented in other cardiovascular diseases [30, 32–35], but these have been seldom examined in AF. Current AF management approaches may be widening the health disparities gap by failing to take into account differences in patient-reported outcomes by these key characteristics [36–39].

A cohort study was conducted to determine individual (sex, age, education level) characteristics that are associated with patient-reported outcomes (quality of life, symptom severity, and emotional and functional status), and if individual characteristics affect the change in patient-reported outcomes over time. We hypothesized that female sex, older age, and low education level as a surrogate of socioeconomic status (SES), would be associated with higher symptom severity and lower quality of life, emotional status and functional status.

## Methods

### Design and setting

The PaTH Clinical Data Research Network (CDRN) initiative aims to use clinical data from electronic medical records and patient reported outcomes to answer questions of clinical importance to patients, providers, and other stakeholders [40]. The research infrastructure provided through the PaTH network provides opportunities to researchers seeking to conduct studies examining variables collected by the PaTH network. PaTH AF is a longitudinal cohort study at four academic medical centers [40]. PaTH AF has two components: 1) a cohort of over 95,302 individuals identified through electronic medical records, this dataset is thus limited to de-identified information from electronic medical records, and 2) a “Consented Cohort” of 953 consented individuals who were recruited from the larger cohort. The PaTH CDRN utilizes a central Institutional Review Board approach which governs the entire process. The institutional review board approved this study (JHU IRB00064600).

### Inclusion and exclusion criteria

Inclusion criteria for the PaTH AF Cohort included: diagnosis of AF by ICD 9–10 codes or electrocardiographic reading, 3 non-emergency department visits since 1/1/2011, age ≥ 18, and ability to speak, read, and understand English. Individuals were excluded if they received the diagnosis of AF within a month of cardiac or abdominal surgery, or a thyroid-related diagnosis, and 12 months before or after prescription for methimazole or propylthioracil. Individuals in the PaTH AF Cohort were identified through electronic medical records using a computable phenotype [41] implementing the inclusion and exclusion criteria.

### Recruitment, screening, and data collection procedures

Participants were recruited for the Consented Cohort to investigate patient-reported outcomes beginning in August 2015. Individuals identified in the larger PaTH AF clinical database were contacted with study invitations through in-person, email, phone, patient portal messaging and post mail techniques. Participants were screened to ensure they were correctly identified as meeting the inclusion and exclusion criteria through an online survey.

At the time of study enrollment, participants completed baseline questionnaires of demographic and health status information, and participants completed surveys on patient-reported outcomes every 6 months following completion of the baseline questionnaire. Data collection was done online. Participants recruited through email and post mail completed surveys through REDCap (Vanderbilt University, TN), and participants who were recruited through the patient portal completed the surveys in MyChart (EPIC software, WI). Either MyChart or REDCap was used to enroll and collect data on patients recruited in-person and by phone based on patient preference.

The cohort completes surveys on patient-reported outcomes at baseline and every 6 months. Healthcare utilization, procedures, medications, diagnoses, body mass index, laboratory data, and demographics are collected through electronic medical records. This analysis consists of participants’ first 3 patient-reported outcome surveys (baseline, 6 months, and 1 year), and electronic medical record data collected through the time of the 1-year post-enrollment survey.

The PaTH network is committed to high quality data and uses the PCORnet Common Data Model, a defined set of health data in a structured, consistent format [40]. The Common Data Model design prioritizes analytic functionality and a parsimonious approach to ensure the electronic medical record data is accurate and consistent across sites. Additionally, each site had a trained research assistant and coordinator to oversee the quality of the data collected via surveys.

### Instruments and variable definitions

Age, sex, education, and marital status were determined by self-report. Comorbidities, body mass index (BMI), and medication prescriptions were determined through electronic medical record data. Comorbidities were measured using the grouped Charlson comorbidities index score, which is calculated using the Deyo version of the Charlson comorbidities index [42, 43]. The grouped Charlson comorbidities index score rates comorbidities on a 0 to 2 scale.

Symptoms and quality of life were measured using the Atrial Fibrillation Effect on QualiTy of Life (AFEQT) [44]. The AFEQT is a disease-specific quality of life instrument consisting of four subscales: symptoms, treatment concerns, daily activities, and treatment satisfaction. The subscales for symptoms, treatment concerns, and daily activities are added together to create an AF-related quality of life score. The AFEQT has high internal consistency (Cronbach’s α: > 0.8) and has been validated in a large, community-based cohort [7, 9, 44, 45]. In this sample, the AFEQT had a Cronbach’s alpha of 0.93. The symptoms subscale consists of 4 items rated on a 7-point Likert scale and contains questions asking patients to report in the past 4 weeks, how much were they bothered by palpitations, an irregular heart beat, a pause in heart activity, and lightheadedness or dizziness as a result of their AF. The symptoms subscale was analyzed separately from the AF-related quality of life total score since patients’ reported symptoms are the primary focus of AF therapies. The symptoms subscale score is formed by calculating the mean score of the questions answered. The AFEQT total scale scores range from 0 to 100, with 0 representing the worst possible quality of life, and 100 representing the best (no impairment due to AF).

Emotional and functional status were measured using the Patient-Reported Outcomes Measurement Information System (PROMIS)-29 emotional distress (anxiety and depression) and physical function domains [46]. The PROMIS initiative was established to develop standardized, reliable, and valid item banks for measuring patient-reported outcomes that are highly accessible to researchers, patients and clinicians in multiple languages and free-of-charge [46–48]. The emotional distress (4 items on anxiety and 4 items on depression) and physical function domains (4 items) are rated on a 5-point Likert-type scale. High internal consistency have been reported for both emotional (Cronbach’s α: > 0.8) [46, 48] and physical function domains (Cronbach’s α: > 0.8) [46, 48]. The raw scores of the anxiety, depression, and functional status domains were converted to a scaled T-score that range from 41 to 79.4 for depression, 40.3 to 81.6 for anxiety, and 22.9 to 56.9 for function using PROMIS scoring guidelines [49]. The scaled T-score is created based on PROMIS scores mean of 50 and standard deviation of 10 in a generalized, referent population: scores 0.5–1.0 standard deviation worse than the mean are interpreted as mild symptoms or impairment, scores 1–2 standard deviations are interpreted as moderate symptoms or impairment, and scores 2 standard deviations or more worse than the mean is interpreted as severe symptoms or impairment.

Rate and rhythm control medication were included as covariates in all final models. Patients were classified as receiving rate control medication or rhythm control medication if they had been prescribed a medication in an outpatient setting in the respective category within 1 year prior to their baseline questionnaire. Medications were categorized based on the American College of Cardiology’s recommendations [50]. Rate control medications included amiodarone, atenolol, carvedilol, diltiazem, metoprolol, and verapamil. Rhythm control medications included amiodarone, dofetilide, dronedarone, flecainide, ibutilide, propafenone, and sotatol. Ablation was classified based on provider billing code CPT 93656 within the past 3 years. Cardioversion was classified based on provider billing codes CPT 92960, 92,961, or ICD-9 99.61 within the past 3 years.

### Statistical methods

We used the statistical software STATA, version 15.0 (StataCorp, College Station, TX), for all analyses. We checked model assumptions of normality before statistical analyses. We calculated descriptive statistics for all variables included in the analysis to examine means, standard deviations, shapes of distributions for continuous variables, and frequencies for categorical variables. Patterns of missing data were assessed for each variable included in the models, and, for variables with greater than 5% missing, missing data was imputed using multiple imputation [51, 52]. We created 100 imputations for each patient-reported outcome (symptoms, AF-related quality of life, anxiety, and depression) since these 13% of these measures were missing at baseline, and 33% of these measures were missing across all 3 survey times (baseline, 6 months, and 1 year). Missing data across patient-reported outcomes did not significantly differ by sex, comorbidities, age, education, or BMI, however, missing data did significantly differ by site. When we compared results using completed-cases only against results using multiple imputation, we found that inferences did not differ across the models (see Appendix). For all of regression analyses described below, we examined multi-collinearity within each set and among all variables by examining the variance inflation factor (VIF). Variables with a high VIF (0.8 or greater), which is indicative of multi-collinearity, were considered for removal from the model. Patient reported health status was collinear with functional status and emotional distress, and thus was removed from the model.

Longitudinal multiple regressions with random effects tested the associations of individual characteristics to patient-reported outcomes over time. The Lagrangian multiplier test determined that random effects was appropriate (*p* < 0.05). Quality of life, symptom severity, emotional status and functional status were each treated as separate dependent variables. Variables were entered as sets into the model with individual characteristics entered in the first step, followed by comorbidities, BMI, and rate and rhythm control medications in the second step. Age in years was divided into 4 categories as follows: 1) > 18 and < 60, 2) > 60 and < 70, 3) > 70 and < 80, and 4) > 80. Site was dummy-coded and included in all models. The Wald test was used to test categorical variables. In the third and final set of models, change in patient-report outcomes over the 6-month intervals by individual characteristics was examined. The interaction of female sex and time, age group and time, and education level and time were each examined in separate models that accounted for comorbidities, BMI, rate control medications, and rhythm control medications.

## Results

### Sample characteristics

Table 1 summarizes demographic data specific to all participants stratified by sex. Our study population (*n* = 953) was 65% male (*n* = 616), 93% white (*n* = 890), and 72 (±10) years old on average. The majority (63%) had at least a college degree. A diagnosis of heart failure was present in 28% of participants (*n* = 364), and the average Charlson Comorbidity Index among participants was 1.61 (±2.08). The majority of the population was overweight (35%) or obese (44%). The patient-reported outcomes of this sample were each lower than the general population mean of 50 [46]: the mean functional status of our population was 47.3 (±8.8), the mean anxiety score of our population was 48.9 (±8.8), and the mean depression score of our sample was 46.9 (±8.1). Participants reported a mean score of 2.21 (±1.40) on the AF symptoms subscale [1–7], which can be interpreted as the participants were on average “a little bothered” by their AF symptoms. On average, the AFEQT total score was 62.72 (25.80) on a scale of 0 to 100, with 0 being the lowest AF-related quality of life.

**Table 1:** Demographic Characteristics by Sex, *N* = 953

	Total %(n)	Female (337)	Male (616)	OR/β (95% CI)
Sex				
Female	35 (337)			
Male	65 (616)			
Race				
White	93 (890)	90 (304)	95 (586)	Ref
Black	5 (47)	8 (27)	3 (20)	**2.61 (1.46, 4.68)**
Other	1 (16)	2 (6)	2 (10)	1.18 (0.42, 3.27)
Age, mean (SD)	71.7 (10)	70.7 (11.3)	72.2 (9.6)	0.98, (0.97, 1.00)
Age Group				
> 18 and < 60	12 (115)	15 (52)	10 (63)	Ref
> 60 and < 70	26 (244)	25 (83)	26 (161)	0.64 (0.41, 1.01)
> 70 and < 80	40 (380)	37 (125)	41 (255)	0.59 (0.39, 0.90)
> 80	22 (214)	23 (77)	23 (137)	0.66 (0.42, 1.05)
Education				
> than college	40 (358)	30 (107)	38 (251)	Ref
College	22 (198)	20 (60)	25 (138)	1.02 (0.70, 1.49)
Some college	23 (200)	27 (81)	23 (119)	**1.60 (1.11, 2.29)**
High School or less	14 (130)	23 (60)	15 (70)	**2.01 (1.33, 3.04)**
Charlson Comorbidity Index				
0	40 (374)	43 (144)	37 (230)	Ref
1	21 (195)	20 (65)	21 (130)	0.82 (0.57, 1.17)
2	39 (369)	36 (122)	40 (247)	0.79 (0.59, 1.07)
Heart Failure				
Present	28 (263)	23 (77)	30 (186)	0.69 (0.51, 0.94)
Not Present	72 (689)	77 (259)	70 (430)	
BMI				
Normal	20 (185)	24 (78)	18 (107)	Ref
Underweight	1 (8)	2 (7)	0 (1)	**9.8 (1.18, 81.53)**
Overweight	35 (324)	28 (93)	39 (231)	**0.56 (0.38, 0.82)**
Obese	44 (408)	46 (152)	43 (256)	0.82 (0.58, 1.16)
Rate Control Medication				
*History of Prescription*				
Yes	52 (492)	50 (168)	53 (324)	
No	48 (461)	50 (169)	47 (292)	0.90 (0.69, 1.17)
*Prescription in the Past Year*				
Yes	16 (158)	15 (51)	17 (107)	
No	83 (799)	85 (286)	83 (513)	0.85 (0.59, 1.23)
Rhythm Control Medication				
*History of Prescription*				
Yes	54 (514)	57 (192)	52 (322)	
No	46 (439)	43 (145)	48 (294)	1.21 (0.92, 1.58)
*Prescription in the Past Year*	28 (267)	31 (106)	26 (161)	
Yes		69 (231)	74 (455)	1.30 (0.97, 1.74)
No	72 (686)			
Quality of Life	62.72 (25.80)	59.87 (25.53)	64.31 (25.84)	**−4.13 (−8.10, −0.17)**
Symptom Severity	2.21 (1.4)	2.42 (1.44)	2.10 (1.38)	**0.32 (0.10, 0.54)**
Functional Status	47.30 (8.8)	45.85 (8.89)	48.10 (8.65)	**−1.80 (−2.99, − 0.60)**
Anxiety	48.93 (8.75)	50.37 (9)	48.12 (8.52)	**1.91 (0.79, 3.02)**
Depression	46.86 (8.08)	47.82 (8.52)	46.31 (7.77)	**1.36 (0.34, 2.36)**
Years since original AF Diagnosis recorded in EMR	1.95 (1.74)	1.83 (1.76)	2.01 (1.73)	0.94 (0.87–1.02)

Signficant results (*p*<0.05) are indicated by bold font

In unadjusted regression models, women were significantly more likely to report poor AF-related quality of life (β − 4.13, 95% CI: -8.10, − 0.17), higher symptom severity (β 0.32, 95% CI: 0.10, 0.54), poor functional status (β − 1.80, 95% CI: -2.99, − 0.60), and higher symptoms of anxiety (β 1.91, 95% CI: 0.79, 3.02) and depression (β 1.36, 95% CI: 0.34, 2.36) at baseline. In unadjusted logistic regression models, women were significantly less likely to have heart failure (OR 0.69, 95% CI 0.51, 0.94), be overweight (OR 0.56, 95% CI 0.38, 0.92) and have a higher level of education (χ2 15.64, *p* < 0.05).

### Patient-reported outcomes by sex

**Fig. 1:**
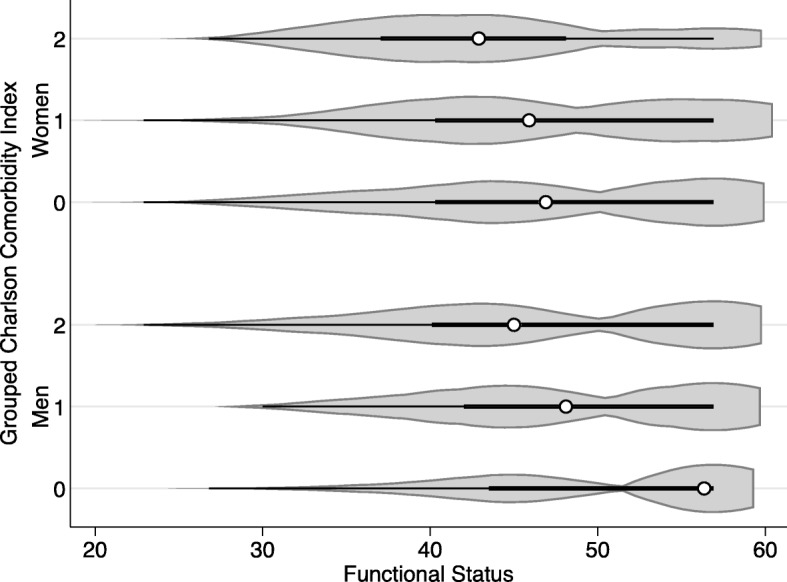
Violin Plot of Functional Status by Comorbidities and Sex

Longitudinal multiple regression results testing individual characteristics associated with patient-reported outcomes are summarized in Table 2. In models including only individual characteristics (sex, education, and age) and patient-reported outcomes, female sex was associated with higher AF symptoms (β 0.19, 95% CI: 0.13, 0.46), anxiety (β 1.79, 95% CI: 0.65, 2.93), and depression (β 1.14, 95% CI: 0.10, 2.18), and lower function status (β − 2.03, 95% CI: -3.22, − 0.85). Female sex was not associated with AF-related quality of life in the individual characteristics model. In longitudinal models adjusting for illness characteristics (BMI, comorbidities, and heart failure) and rate and rhythm control medication prescription, female sex was significantly associated with higher symptoms of AF (β 0.14, 95% CI: 0.02, 0.25), anxiety (β 1.90, 95% CI: 0.7, 3.10) and depression (β 1.39, 95% CI: 0.27, 2.50), and poorer AF-related quality of life (β − 2.89, 95% CI: -5.74, − 0.03) and functional status (β − 1.99, 95% CI: -3.19, − 0.80). While women tended to have poorer patient-reported outcomes, female sex was not associated with illness characteristics on patient-reported outcomes (Fig. 1).

**Table 2:** Longitudinal Data Analysis: Patient Report Outcomes by Individual Characteristics (Individual Model)

	Symptoms (β, 95% CI)	Anxiety (β, 95% CI)	Depression (β, 95% CI)	Function (β, 95% CI)	AF-related QOL (β, 95% CI)
Age Group					
> 18 and < 60	Ref	Ref	Ref	Ref	Ref
> 60 and < 70	**−0.36 (−0.62, −0.09)**	**−3.10 (−4.90, −1.29)**	**−2.47 (− 4.08, − 0.86)**	−1.33 (− 3.16, 0.49)	2.96 (− 2.21, 8.12)
> 70 and < 80	**− 0.51 (− 0.76, − 0.26)**	**− 3.41 (−5.17, − 1.66)**	**−2.69 (− 4.27, − 1.12)**	**−2.68 (− 4.50, − 0.87)**	2.44 (− 2.61, 7.49)
> 80	**−0.59 (− 0.87, − 0.31)**	**−4.63 (− 6.56, − 2.71)**	**− 3.40 (− 5.12, − 1.67)**	**− 5.98 (−7.94, − 4.02)**	1.93 (− 3.49, 7.35)
Female Sex	**0.19 (0.13, 0.46)**	**1.79 (0.65, 2.93)**	**1.14 (0.10, 2.18)**	**−2.03 (− 3.22, − 0.85)**	−2.28 (− 540, 0.85)
Education					
> than college	Ref	Ref	Ref	Ref	Ref
College	0.11 (− 0.08, 0.31)	−0.06 (− 1.45, 1.34)	0.52 (− 0.76, 1.79)	**−2.00 (− 3.51, − 0.49)**	−1.08 (− 4.96, − 2.79)
Some college	**0.32 (0.12, 0.52)**	**1.73 (0.33, 3.13)**	**1.11 (0.15, 2.38)**	**−2.50 (− 3.99, − 1.01)**	**−5.34 (−9.19, − 1.49)**
High School or less	0.10 (−0.13, 0.32)	− 0.02 (− 1.64, 1.60)	0.83 (− 0.63, 2.29)	**−2.47 (− 4.17, − 0.76)**	−3.63 (− 7.98, 0.72)

Signficant results (*p*<0.05) are indicated by bold font

In models accounting for education, age, comorbidities, BMI, and rate and rhythm control medication, the association of female sex and patient-reported outcomes varied over time. Women reported significantly higher AF symptoms at baseline (β 0.29, 95%CI: 0.08, 0.50). The change in AF symptoms over time did not differ across sexes. Women reported higher symptoms of anxiety at baseline (β 2.08, 95% CI: 0.76, 3.40), 6 months (β 2.28, 95% CI: 0.77, 3.80) and 1 year (β 1.82, 95% CI: 0.32, 3.32). At baseline, women reported significantly higher symptoms of anxiety (β 1.44, 95% CI: 0.25, 2.63) but the change over time was non-significant between sexes. Women reported poor functional status at baseline (β − 2.23, 95% CI: -3.52, − 0.94), 6 months (β − 2.94, 95% CI: -4.39, − 1.48) and 1 year (β − 3.18, 95% CI: -4.62, − 1.75). While women reported poorer AF-related quality of life at baseline (β − 4.12, 95% CI: -8.10, − 0.14), the change over time in AF-related quality of life was not significantly different across sexes.

### Patient-reported outcomes by age

**Fig. 2:**
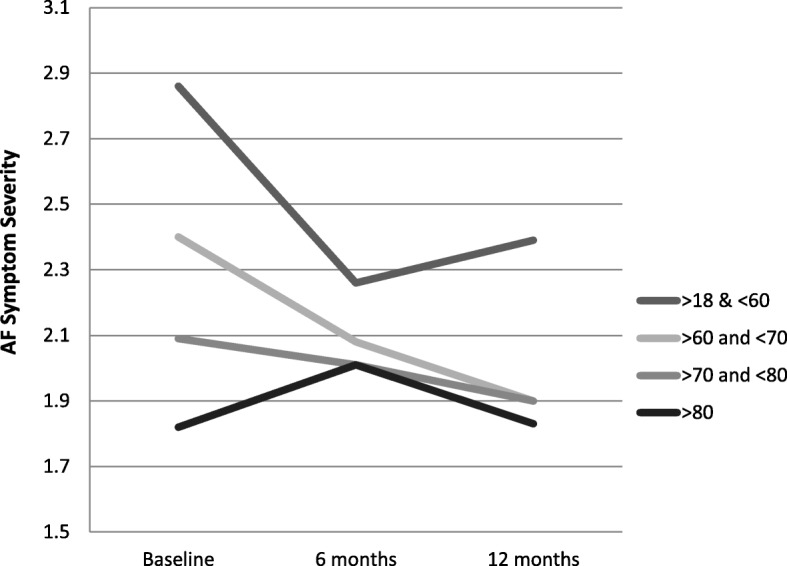
Age and Reported Symptom Severity Over One Year

In longitudinal multiple regression models including only individual characteristics (age, sex, and education) with patient-reported outcomes, there was a significant decrease in symptoms of anxiety, depression, and AF with each increase in age category, from age > =60 and < 70, age > =70 and < 80, and age > =80 (Fig. 2). There was no association between AF-related quality of life and age. Compared to individuals under age 60, individuals between the ages of 70 and 80 (β − 2.68, 95% CI: -4.50, − 0.87) and older than 80 (β − 5.98, 95% CI: -7.94, − 4.02) reported poorer functional status.

In longitudinal models adjusting for illness characteristics (BMI, comorbidities, and heart failure) and rate and rhythm control medication prescription, there remained a decrease in symptoms of anxiety, depression, and AF with each increase in age category, from age > =60 and < 70, age > =70 and < 80, and age > =80. There remained no association between AF-related quality of life and age. Compared to individuals under age 60, individuals between the ages of 70 and 80 (β − 2.30, 95% CI: -4.11, − 0.50) and older than 80 (β − 6.76, 95% CI: -8.76, − 4.77) reported poorer functional status.

In models accounting for education, age, comorbidities, BMI, and rate and rhythm control medication, older age groups reported significantly lower symptoms of AF, anxiety, and depression compared to individuals under age 60 at baseline, 6 months, and 12 months (Table 3). With the exception of adults between the ages of 60 to 70 at baseline, older age groups reported poorer functional status at baseline, 6 months, and 12 months. Individuals between the ages of 60 to 70 reported better AF-related quality of life at 12 months (β 9.54, 95% CI: 2.57, 16.52**)** compared to individuals under the age of 60. AF-related quality of life at baseline and the change in AF-related quality of life at 6 months and 12 months was not significantly different in individuals over the age of 70 compared to individuals under the age of 60.

**Table 3:** Longitudinal Data Analysis: Patient Report Outcomes Changes Over One year by Individual Characteristics^a^

	Symptoms (β, 95% CI)	Anxiety (β, 95% CI)	Depression (β, 95% CI)	Function (β, 95% CI)	AF-related QOL (β, 95% CI)
Age Group					
> 18 and < 60	Ref	Ref	Ref	Ref	Ref
> 60 and < 70					
Baseline	**−0.44 (− 0.78, − 0.12)**	**− 3.13 (− 5.19, 1.06)**	**−1.93 (− 4.08, − 0.86)**	−1.04 (− 2.98, 0.90)	1.38 (− 5.11, 7.88)
6 month	**−0.73 (− 1.06, − 0.39)**	**−2.75 (− 4.94, − 0.56)**	**−3.12 (− 4.27, − 1.12)**	**−1.82 (− 3.88, 0.24)**	2.16 (− 4.72, 9.04)
12 month	**−0.90 (− 1.24, − 0.56)**	**−3.63 (− 5.82, − 1.44)**	**− 2.93 (− 5.12, − 1.67)**	**−1.92 (− 3.97, 0.14)**	**9.54 (2.57, 16.52)**
> 70 and < 80					
Baseline	**−0.70 (− 1.01, − 0.38)**	**− 4.07 (− 6.05, − 2.09)**	**−2.74 (− 4.42, − 0.96)**	**−2.16 (− 4.07, − 0.25)**	1.10 (−5.32, 7.51)
6 month	**−0.81 (− 1.14, − 0.49)**	**−3.05 (− 5.14, − 0.96)**	**−2.95 (− 4.82, − 1.09)**	**− 3.22 (− 5.22, − 1.23)**	5.95 (− 0.66, 12.55)
12 month	**−0.87 (−1.19, − 0.54)**	**−3.68 (− 5.77, − 1.59)**	**− 2.74 (− 4.60, − 0.88)**	**− 3.71 (− 5.70, − 1.72)**	2.42 (− 4.24, 9.08)
> 80					
Baseline	**−0.80 (− 1.16, − 0.45)**	**−5.06 (− 7.31, − 2.81)**	**−3.79 (− 5.80, − 1.78)**	**− 6.24 (− 8.36, − 4.12)**	0.27 (− 6.72, 7.25)
6 month	**−0.77 (− 1.13, − 0.40)**	**−4.59 (− 7.02, − 2.17)**	**− 2.67 (− 4.82, − 0.51)**	**−7.32 (− 9.60, − 5.04)**	2.40 (− 5.12, 9.92)
12 month	**− 0.84 (− 1.21, − 0.48)**	**−4.46 (− 6.80, − 2.12)**	**− 2.94 (− 5.02, − 0.85)**	**−7.65 (− 9.88, − 5.41)**	−1.23 (− 8.70, 6.25)
Female Sex					
Baseline	**0.29 (0.13, 0.45)**	**2.08 (0.76, 3.40)**	**1.44 (0.25, 2.63)**	**−2.23 (− 3.52, − 0.94)**	**− 4.08 (− 8.06, − 0.10)**
6 Month	0.15 (− 0.07, 0.36)	**2.28 (0.77, 3.80)**	0.66 (−0.69, 2.01)	**−2.94 (− 4.39, − 1.48)**	0.17 (− 4.30, 4.64)
12 Month	0.10 (− 0.05, 0.26)	**1.82 (0.32, 3.32)**	1.23 (− 0.11, 2.57)	**− 3.18 (− 4.62, − 1.75)**	0.29 (− 4.21, 4.80)
Education					
> than college	Ref	Ref	Ref	**Ref**	Ref
College					
Baseline	0.06 (−0.19, 0.32)	0.12 (− 1.48, 1.72)	0.26 (− 1.18, 1.70)	**− 1.66 (− 3.26, − 0.06)**	−0.66 (− 4.50, 3.18**)**
6 month	− 0.01 (− 0.28, 0.25)	1.10 (− 0.70, 2.90)	0.91 (− 0.69, 2.51)	**− 3.41 (− 5.16, − 1.66)**	1.78 (− 3.79, 7.36)
12 month	− 0.09 (− 0.36, 0.18)	0.40 (− 1.44, 2.25)	0.64 (− 1.00, 2.28)	**− 3.28 (− 5.05, − 1.52)**	1.66 (− 4.06, 7.37)
Some college					
Baseline	**0.42 (0.17, 0.68)**	**1.86 (0.26, 3.45)**	1.40 (−0.04, 2.84)	**− 2.04 (− 3.63, − 0.44)**	**−4.41 (− 8.25, − 0.57)**
6 month	0.02 (− 0.25, 0.29)	**2.71 (0.82, 4.61)**	0.67 (− 1.01, 2.35)	**− 2.66 (− 4.45, − 0.88)**	−1.53 (− 7.19, 4.13)
12 month	−0.04 (− 0.31, 0.23)	**2.13 (0.33, 3.94)**	0.73 (− 0.88, 2.34)	**− 2.90 (− 4.63, − 1.16)**	−1.39 (− 7.05, 4.27)
High School or less					
Baseline	0.02 (− 0.26, 0.31)	0.86 (−1.00, 2.71)	1.08 (− 0.58, 2.74)	**− 2.86 (− 4.68, − 1.04)**	−3.93 (− 6.88, 1.81)
6 month	− 0.04 (− 0.35, 0.26)	1.30 (−0.91, 3.50)	0.35 (−1.61, 2.31)	**−3.38 (− 5.47, − 1.29)**	−0.87 (− 7.15, 5.41)
12 month	−0.13 (− 0.44, 0.18)	0.69 (−1.47, 2.85)	0.94 (− 0.98, 2.86)	**−3.63 (− 5.69, − 1.58)**	1.33 (− 5.14, 7.81)

^a^Model adjusted for the Charlson Comorbidity Index, body mass index, and rate and rhythm medication prescription

Signficant results (*p*<0.05) are indicated by bold font

### Patient reported outcomes by education level

In longitudinal multiple regression models including only individual characteristics (age, sex, and education) with function, anxiety, and depression, functional status decreased with education level. Compared to individuals with greater than a college education, Individuals with a college education (β − 2, 95% CI: -3.51, − 0.49), individuals with some college (β − 2.5, 95% CI: -3.99, − 1.01), and individuals with high school or less (β − 2.47, 95% CI: -4.17, − 0.76) reported poorer functional status. Individuals with some college education reported higher symptoms of AF (β 0.14, 95% CI 0.01, 0.27), anxiety (β 1.73, 95% CI: 0.33, 3.13), and depression (β 1.11, 95% CI: 0.15, 2.38) compared to individuals with greater than a college education. Individuals with some college education reported poorer AF-related quality of life (β − 5.34, 95% CI: -9.19, − 1.49) than individuals with greater than a college education.

In longitudinal multiple regression models adjusting for illness characteristics and rate and rhythm control medication prescription, there was no association between education level and symptoms of depression and AF-related quality of life. Compared to individuals with greater than a college education, Individuals with a college education (β − 1.56, 95% CI: -3.04, − 0.09), individuals with some college (β − 1.77, 95% CI: -3.23, − 0.31), and individuals with high school or less (β − 2.66, 95% CI: -4.35, − 0.98) reported poorer functional status. Individuals with some college education reported higher symptoms of AF (β 0.32, 95% CI 0.12, 0.52) and anxiety (β 1.64, 95% CI: 0.19, 3.09) compared to individuals with greater than a college education.

In models accounting for education, age, comorbidities, BMI, and rate and rhythm control medication, individuals with lower levels of education reported significantly poorer functional status baseline, 6 months, and 12 months compared to individuals with greater than a college education. Individuals with some college reported higher symptoms of AF at baseline (β 0.42, 95% CI: 0.17, 0.68) and anxiety at baseline (β 1.86, 95% CI: 0.26, 3.45), at 6 months (β 2.71, 95% CI: 0.82, 4.61), and 12 months (β 2.13, 95% CI: 0.33, 3.94), and poor AF-related quality of life at baseline (β − 4.41, 95% CI: -8.25, − 0.57) compared to individuals with greater than a college education.

### Patient-reported outcomes by site, illness Effects, and rate and rhythm control medication

Site, BMI, comorbidities, and rate and rhythm control medication prescription in the past year were included as covariates across all final models. Site was associated with anxiety (β − 1.38, 95% CI: -1.94, − 0.82), functional status (β 0.96, 95% CI: 0.39, 1.53), and (β 3.80, 95% CI: 1.79, 5.82). BMI was associated with poorer AF-related quality of life (β 0.58, 95% CI: -0.83, − 0.33) and higher symptoms of depression (β 0.09, 95% CI: 0.01, 0.18). Comorbidities were associated with poorer functional status (β − 1.04, 95% CI: -1.63, − 0.44). Rate medication was associated with poorer functional status (β − 2.16, 95% CI: -3.44, − 0.87) and higher symptoms of depression (β − 1.41, 95% CI: 0.11, 2.70). Rhythm control medication was association with higher symptoms of AF (β 0.35, 95% CI: 0.19, 0.52).

## Discussion

In this multi-institutional, longitudinal cohort of 953 individuals with AF, women, younger adults, and individuals with a low level of education had comparatively poorer patient-reported outcomes. The association between sex, age, education and patient-reported outcomes remained over time and after adjusting for illness characteristics.

Women were more likely to report AF symptoms and poor AF-related quality of life than men. While previous studies have substantiated this association between sex and AF symptoms and quality of life [28, 53], cohort studies have found that women are less likely to receive rhythm control treatments that reduce symptom severity [54–56]. Symptoms of anxiety and depression were significantly more prevalent in women. Previous studies have found an association between depression and anxiety and AF symptoms [57, 58], and qualitative evidence that stress precedes symptomatic AF episodes [12, 59]. It is possible that the higher symptoms of anxiety and depression among women in this sample contribute to higher AF symptom severity.

Younger adults in this sample reported higher symptoms of AF, anxiety, and depression. AF is rare in younger adults, men ages 75 to 79 have double the prevalence rate compared with men 65 to 69, and more than 5 times the prevalence rate of men 55 to 59 [1]. The limited data on the association between age and symptoms in AF is conflicting. Gehi and colleagues similarly found that younger age was associated with higher symptoms [45], and the ORBIT-AF investigators similarly found that younger adults were more likely to have poor AF-related quality of life [53]. Acute life stress is associated with the development of AF [60]. The interplay between anxiety, depression, and symptoms is important to consider in the association of age and AF symptoms. It is possible that younger adults with AF may have had poor patient-reported outcomes prior to the diagnosis of AF and that the association is mediated by other unmeasured confounders.

Our findings of poor AF-related quality of life and higher AF symptom severity at lower levels of education substantiate previous findings [36]. Education level is a social determinant and is linked to increased risk of poor cardiovascular outcomes [35]. Individuals with a low education level are more likely to have poor health literacy and less likely to engage in preventive health [61]. These factors may lead to less educated individuals receiving disparate treatment for rate and rhythm control, which in turn leads to poor AF-related quality of life and increased AF symptoms.

A key strength of this study is the use of PROMIS measures. The PROMIS measures were created to address the need in the clinical research community for a rigorously tested patient reported outcome tool that addresses the lack of standardization in patient-reported outcomes [46, 48]. The PROMIS measures are rigorously validated, reliable, free, available in multiple languages, and simple to score in interpret, thus they are practical for research teams to adapt as opposed to more costly instruments [47, 48, 62]. The use of the PROMIS-29 in this manuscript allows clinicians, researchers, and patients to compare the impact of AF on patient-reported outcomes to other diseases, and the general population.

### Limitations

These data represent observations from a multi-institutional cohort study. Participation in this study is voluntary and, thus, biases exist in patient selection, participating sites, and reporting. We performed multiple imputation to account for reporting bias due to missing data, and found that there was no difference in our conclusions when we examined completed cases only. Despite the use of multiple recruitment methods, the study sample was overwhelmingly white. The larger PaTH clinical database of individuals with AF was majority white (90%), which may be reflective of the population in treatment for AF [37, 39]. This pattern is consistent throughout AF clinical trials [37], there is a lower prevalence of this disease among black and Hispanic populations which adds to the challenge of enrolling a racially diverse group of patients. A further limitation is that we did not measure prior physical activity. There is evidence that endurance athletes are at a heightened risk for atrial fibrillation [63, 64], and accounting for prior athletic activity may have added to our understanding of the differences in atrial fibrillation experience by individual characteristics.

Importantly, this study relies on electronic medical record data for information on rate and rhythm control medication, BMI, and comorbidities. At least 3 non-inpatient visits in the 4 years prior to study enrollment was inclusion criteria to ensure enrolled participants had adequate and accurate electronic medical record data available. However, changes in medications, new diagnoses, and weight fluctuations may have occurred at visits to health systems not included in the PaTH network in parallel to the time of this study, and thus not been captured in this dataset. Site was included as a variable in all models to adjust for differences in both EMR data collection across institutions, and varying patient populations. Site was statistically significant across all models, which highlights the importance of taking site into consideration in multi-institutional research.

## Conclusion

There is great variability in symptom experience and patient-reported outcomes among AF patients. AF symptoms largely guide therapy decisions for rate and rhythm control strategies, yet AF therapy guidelines do not take individual characteristics into account. We have shown that in a multi-institutional cohort of AF patients, individuals with lower education, women, and younger adults are more likely to report poor AF-related quality of life, anxiety, and AF symptoms. Our findings highlight the need to be aware of important differences based on sex, age and education level when communicating with patients and making clinical decisions regarding treatment. Taking these important sociodemographic factors into account when communicating and creating treatment plans may improve patient-reported outcomes among individuals with AF.

## Appendix

**Table 4:** Longitudinal Data Analysis: Patient Report Outcomes by Individual Characteristics – Completers Only Analysis for Comparison to Multiple Imputation Results

	Symptoms (β, 95% CI)	Anxiety (β, 95% CI)	Depression (β, 95% CI)	Function (β, 95% CI)	AF-related QOL (β, 95% CI)
Age Group					
> 18 and < 60	Ref	Ref	Ref	Ref	Ref
> 60 and < 70	**− 0.32 (− 0.57, − 0.07)**	**− 2.86 (− 4.72, − 1.02)**	**−2.71 (− 4.38, − 1.04)**	−1.50 (− 3.38, 0.36)	2.10 (− 3.01, 7.22)
> 70 and < 80	**− 0.46 (− 0.71, − 0.22)**	**− 3.98 (− 5.79, − 2.17)**	**− 2.98 (− 4.64, − 1.33)**	**− 2.24 (− 4.07, − 0.41)**	1.31 (− 3.67, 6.28)
> 80	**−0.49 (− 0.77, − 0.22)**	**−4.57 (− 6.54, − 2.61)**	**−3.45 (− 5.17, − 1.62)**	**−6.17 (− 8.16, − 4.18)**	− 0.33 (− 5.93, 5.28)
Female Sex	**0.30 (0.15, 0.45)**	**1.77 (0.63, 2.90)**	**1.17 (0.12, 2.23)**	**−2.07 (− 3.24, − 0.89)**	−2.51 (− 5.61, 0.58)
Education					
> than college	Ref	Ref	Ref	**Ref**	Ref
College	0.12 (− 0.07, 0.32)	0.04 (− 1.38, 1.47)	0.45 (− 0.87, 1.78)	**− 1.94 (− 3.40, − 0.49)**	0.11 (− 3.71, 4.16)
Some college	0.38 (−0.18, 0.57)	**1.86 (0.43, 3.28)**	1.02 (−0.30, 2.35)	**−1.61 (− 3.08, − 0.15)**	−4.55 (− 8.47, 0.63)
High School or less	0.11 (− 0.11, 0.33)	−0.29 (− 1.94, 1.36)	0.48 (− 1.06, 2.01)	**−1.87 (− 3.58, − 0.16)**	−1.85 (− 6.21, 2.50)

^a^Model adjusted for thne Charlson Comorbidity Index, body mass index, and rate and rhythm medication prescription

## Abbreviations

AF: Atrial fibrillation
AFEQT: Atrial fibrillation effect on quality of life
CDRN: Clinical data research network
ICD: International classification of diseases
PaTH: Pennsylvania, Penn, Temple, and Hopkins Path towards a learning health system
PROMIS: Patient reported outcome measurement information system
SES: Socioeconomic status

## References

[1] Chugh SS, Havmoeller R, Narayanan K (2014). Worldwide epidemiology of atrial fibrillation: a global burden of disease 2010 study. Circulation.

[2] Go AS, Hylek EM, Phillips KA (2001). Prevalence of diagnosed atrial fibrillation in adults: national implications for rhythm management and stroke prevention: the AnTicoagulation and risk factors in atrial fibrillation (ATRIA) study. JAMA.

[3] Naccarelli GV, Varker H, Lin J, Schulman KL (2009). Increasing prevalence of atrial fibrillation and flutter in the United States. Am J Cardiol.

[4] Coromilas J (2004). Obesity and atrial fibrillation: is one epidemic feeding the other?. JAMA J Am Med Assoc.

[5] Wang TJ, Parise H, Levy D (2004). Obesity and the risk of new-onset atrial fibrillation. JAMA.

[6] Wong CX, Brooks AG, Lau DH (2012). Factors associated with the epidemic of hospitalizations due to atrial fibrillation. Am J Cardiol.

[7] Rienstra M, Lubitz SA, Mahida S (2012). Symptoms and functional status of patients with atrial fibrillation: state of the art and future research opportunities. Circulation.

[8] Freeman JV, Simon DN, Go AS (2015). Association between atrial fibrillation symptoms, quality of life, and patient outcomes: results from the outcomes registry for better informed treatment of atrial fibrillation (ORBIT-AF). Circ Cardiovasc Qual Outcomes.

[9] Reynolds MR, Morais E, Zimetbaum P (2010). Impact of hospitalization on health-related quality of life in atrial fibrillation patients in Canada and the United States: results from an observational registry. Am Heart J.

[10] Vermond RA, Crijns HJGM, Tijssen JGP (2014). Symptom severity is associated with cardiovascular outcome in patients with permanent atrial fibrillation in the RACE II study. Europace.

[11] MaCrae CA (2009). Editorial: symptoms in atrial fibrillation; why keep score?. Circ Arrhythmia Electrophysiol.

[12] McCabe PJ, Barnason SA (2012). Illness perceptions, coping strategies, and symptoms contribute to psychological distress in patients with recurrent symptomatic atrial fibrillation. J Cardiovasc Nurs.

[13] Sears SF, Serber ER, Alvarez LG, Schwartzman DS, Hoyt RH, Ujhelyi MR (2005). Understanding atrial symptom reports: objective versus subjective predictors. Pacing Clin Electrophysiol.

[14] Patel N, Chung EH, Mounsey JP, Schwartz JD, Pursell I, Gehi AK (2014). Effectiveness of atrial fibrillation monitor characteristics to predict severity of symptoms of atrial fibrillation. Am J Cardiol.

[15] Patten M, Maas R, Karim A, Müller HW, Simonovsky R, Meinertz T (2006). Event-recorder monitoring in the diagnosis of atrial fibrillation in symptomatic patients: subanalysis of the SOPAT trial. J Cardiovasc Electrophysiol.

[16] Quirino G, Giammaria M, Corbucci G (2009). Diagnosis of paroxysmal atrial fibrillation in patients with implanted pacemakers: relationship to symptoms and other variables. Pacing Clin Electrophysiol.

[17] Cosedis Nielsen J, Johannessen A, Raatikainen P (2012). Radiofrequency ablation as initial therapy in paroxysmal atrial fibrillation. N Engl J Med.

[18] January CT, Wann LS, Alpert JS, et al. Guideline for the Management of Patients with Atrial Fibrillation a Report of the. Am Coll Cardiol. 2014. 10.1161/CIR.0000000000000041/-/DC1.The. 10.1016/j.jacc.2014.03.022 24685669

[19] Prystowsky EN, Padanilam BJ, Fogel RI (2015). Treatment of atrial fibrillation. JAMA.

[20] Efremidis M, Letsas KP, Lioni L (2014). Association of quality of life, anxiety, and depression with left atrial ablation outcomes. Pacing Clin Electrophysiol.

[21] Bai Y, Bai R, Wu J (2015). Differences in quality of life between atrial fibrillation patients with low stroke risk treated with and without catheter ablation. J Am Heart Assoc.

[22] Sang C-H, Chen K, Pang X-F (2013). Depression, anxiety, and quality of life after catheter ablation in patients with paroxysmal atrial fibrillation. Clin Cardiol.

[23] Bulková V, Fiala M, Havránek S (2014). Improvement in quality of life after catheter ablation for paroxysmal versus long-standing persistent atrial fibrillation: a prospective study with 3-year follow-up. J Am Heart Assoc.

[24] Mohanty S, Santangeli P, Mohanty P (2014). Catheter ablation of asymptomatic longstanding persistent atrial fibrillation: impact on quality of life, exercise performance, arrhythmia perception, and arrhythmia-free survival. J Cardiovasc Electrophysiol.

[25] Atwood JE, Myers JN, Tang XC, Reda DJ, Singh SN, Singh BN (2007). Exercise capacity in atrial fibrillation: a substudy of the Sotalol-amiodarone atrial fibrillation efficacy trial (SAFE-T). Am Heart J.

[26] Ha ACT, Breithardt G, Camm AJ, et al. Health-related quality of life in patients with atrial fibrillation treated with rhythm control versus rate control: insights from a prospective international registry (registry on cardiac rhythm disorders assessing the control of atrial fibrillation: REC. Circ Cardiovasc Qual Outcomes 2014;7(6):896–904. doi:10.1161/HCQ.0000000000000011. 10.1161/HCQ.0000000000000011 25387780

[27] Steg PG, Alam S, C-EE C (2012). Symptoms, functional status and quality of life in patients with controlled and uncontrolled atrial fibrillation: data from the RealiseAF cross-sectional international registry. Heart.

[28] Gleason KT, Nazarian S, Dennison Himmelfarb CR (2017). Atrial fibrillation symptoms and sex, Race, and psychological distress. J Cardiovasc Nurs.

[29] Narasimha D, Curtis AB (2015). Sex differences in utilisation and response to implantable device therapy. Arrhythm Electrophysiol Rev.

[30] Zusterzeel R, Selzman KA, Sanders WE (2014). Cardiac resynchronization therapy in women: US Food and Drug Administration meta-analysis of patient-level data. JAMA Intern Med.

[31] Kim ESH, Menon V (2009). Status of women in cardiovascular clinical trials. Arterioscler Thromb Vasc Biol.

[32] Rich MW, Chyun DA, Skolnick AH (2016). Knowledge gaps in cardiovascular Care of the Older Adult Population: a scientific statement from the American Heart Association, American College of Cardiology, and American Geriatrics Society. Circulation.

[33] Maddox TM, Reid KJ, Spertus JA (2008). Angina at 1 year after myocardial infarction: prevalence and associated findings. Arch Intern Med.

[34] Weaver WD, White HD, Wilcox RG, et al. Comparisons of characteristics and outcomes among women and men with acute myocardial infarction treated with thrombolytic therapy. GUSTO-I investigators. JAMA. 1996;275(10):777–82. http://ovidsp.ovid.com/ovidweb.cgi?T=JS&PAGE=reference&D=med4&NEWS=N&AN=8598594. 8598594

[35] Havranek EP, Mujahid MS, Barr DA (2015). Social determinants of risk and outcomes for cardiovascular disease: a scientific statement from the American Heart Association. Circulation.

[36] Goli NM, Thompson T, Sears SF (2012). Educational attainment is associated with atrial fibrillation symptom severity. Pacing Clin Electrophysiol PACE.

[37] Golwala H, Jackson LR, Simon DJN (2016). Racial/ethnic differences in atrial fibrillation symptoms, treatment patterns, and outcomes: insights from outcomes registry for better informed treatment for atrial fibrillation registry. Am Heart J.

[38] Mensah GA, Mokdad AH, Ford ES, Greenlund KJ, Croft JB (2005). State of disparities in cardiovascular health in the United States. Circulation.

[39] Bhave PD, Lu X, Girotra S, Kamel H, Vaughan Sarrazin MS (2015). Race- and sex-related differences in care for patients newly diagnosed with atrial fibrillation. Heart Rhythm.

[40] Amin W, Tsui F, Borromeo C (2014). PaTH: towards a learning health system in the mid-Atlantic region. J Am Med Informatics Assoc.

[41] Geva A, Gronsbell JL, Cai T (2017). A computable phenotype improves cohort ascertainment in a pediatric pulmonary hypertension registry. J Pediatr.

[42] Charlson ME, Pompei P, Ales KL, MacKenzie CR (1987). A new method of classifying prognostic comorbidity in longitudinal studies: development and validation. J Chronic Dis.

[43] Deyo RA, Cherkin DC, Ciol MA (1992). Adapting a clinical comorbidity index for use with ICD-9-CM administrative databases. J Clin Epidemiol.

[44] Spertus J, Dorian P, Bubien R (2011). Development and validation of the atrial fibrillation effect on QualiTy-of-life (AFEQT) questionnaire in patients with atrial fibrillation. Circ Arrhythmia Electrophysiol.

[45] Gehi AK, Sears S, Goli N (2012). Psychopathology and symptoms of atrial fibrillation: implications for therapy. J Cardiovasc Electrophysiol.

[46] Cella D, Riley W, Stone A (2010). The patient-reported outcomes measurement information system (PROMIS) developed and tested its first wave of adult self-reported health outcome item banks: 2005-2008. J Clin Epidemiol.

[47] Stone AA, Broderick JE, Junghaenel DU, Schneider S, Schwartz JE. PROMIS fatigue, pain intensity, pain interference, pain behavior, physical function, depression, anxiety, and anger scales demonstrate ecological validity. J Clin Epidemiol. 2016. 10.1016/j.jclinepi.2015.08.029. 10.1016/j.jclinepi.2015.08.029 26628334

[48] Riley WT, Rothrock N, Bruce B (2010). Patient-reported outcomes measurement information system (PROMIS) domain names and definitions revisions: further evaluation of content validity in IRT-derived item banks. Qual Life Res.

[49] Pilkonis PA, et al. Item banks for measuring emotional distress from the patient-reported outcomes measurement information system (PROMIS®): Depression, anxiety, and anger. Assessment. 2011. 10.1177/1073191111411667. PMC3153635 21697139

[50] January CT, et al. 2014 AHA/ACC/HRS Guideline for the Management of Patients With Atrial Fibrillation: Executive Summary. Circulation. 2014. 10.1161/cir.0000000000000040.

[51] Bounthavong M, Watanabe JH, Sullivan KM (2015). Approach to addressing missing data for electronic medical records and pharmacy claims data research. Pharmacotherapy.

[52] Janssen KJM, Donders ART, Harrell FE (2010). Missing covariate data in medical research: to impute is better than to ignore. J Clin Epidemiol.

[53] Randolph TC, Simon DN, Thomas L (2016). Patient factors associated with quality of life in atrial fibrillation. Am Heart J.

[54] Schnabel RB, Pecen L, Ojeda FM (2017). Gender differences in clinical presentation and 1-year outcomes in atrial fibrillation. Heart.

[55] Dagres N, Nieuwlaat R, Vardas PE (2007). Gender-related differences in presentation, treatment, and outcome of patients with atrial fibrillation in Europe. A Report From the Euro Heart Survey on Atrial Fibrillation. J Am Coll Cardiol.

[56] Lip GYH, Laroche C, Boriani G (2014). Sex-related differences in presentation, treatment, and outcome of patients with atrial fibrillation in Europe: a report from the euro observational research Programme pilot survey on atrial fibrillation. Europace.

[57] Thompson TS, Barksdale DJ, Sears SF, Mounsey JP, Pursell I, Gehi AK (2014). The effect of anxiety and depression on symptoms attributed to atrial fibrillation. Pacing Clin Electrophysiol.

[58] Kupper N, van den Broek KC, Widdershoven J, Denollet J. Subjectively reported symptoms in patients with persistent atrial fibrillation and emotional distress. Front Psychol. 2013;4. 10.3389/fpsyg.2013.00192. 10.3389/fpsyg.2013.00192 PMC3634051 23630509

[59] McCabe PJ, Schad S, Hampton A, Holland DE (2008). Knowledge and self-management behaviors of patients with recently detected atrial fibrillation. Hear Lung J Acute Crit Care.

[60] Mattioli AV, Bonatti S, Zennaro M, Mattioli G (2005). The relationship between personality, socio-economic factors, acute life stress and the development, spontaneous conversion and recurrences of acute lone atrial fibrillation. Europace.

[61] Bennett IM, Chen J, Soroui JS, White S (2009). The contribution of health literacy to disparities in self-rated health status and preventive health behaviors in older adults. Ann Fam Med.

[62] Hinchcliff ME, Beaumont JL, Carns MA, et al. Longitudinal evaluation of PROMIS-29 and FACIT-dyspnea short forms in systemic sclerosis. J Rheumatol. 2015. 10.3899/jrheum.140143. 10.3899/jrheum.140143 PMC4480645 25362656

[63] Brugger N, Krause R, Carlen F, et al. Effect of lifetime endurance training on left atrial mechanical function and on the risk of atrial fibrillation. Int J Cardiol. 2014. 10.1016/j.ijcard.2013.11.032. 10.1016/j.ijcard.2013.11.032 24342396

[64] Wilhelm M. Atrial fibrillation in endurance athletes. Eur J Prev Cardiol. 2013. 10.1177/2047487313476414. 10.1177/2047487313476414 23610454

